# Long‐term behavioural outcomes after paediatric convulsive status epilepticus: a population‐based cohort study

**DOI:** 10.1111/dmcn.13636

**Published:** 2017-12-10

**Authors:** Marina M Martinos, Suresh Pujar, Christopher Gillberg, Mario Cortina‐Borja, Brian G R Neville, Michelle De Haan, Rod C Scott, Richard F M Chin

**Affiliations:** ^1^ Developmental Neurosciences Programme UCL Great Ormond Street Institute of Child Health London UK; ^2^ Gillberg Neuropsychiatry Center University of Gothenburg Gothenburg Sweden; ^3^ Population, Policy and Practice Programme UCL Great Ormond Street Institute of Child Health London UK; ^4^ Department of Neurological Sciences University of Vermont Burlington VT USA; ^5^ Muir Maxwell Epilepsy Center University of Edinburgh Edinburgh UK

## Abstract

**Aim:**

To describe behavioural and psychiatric outcomes of children within 10 years of convulsive status epilepticus (CSE).

**Method:**

Children originally identified by the population‐based North London Convulsive Status Epilepticus in Childhood Surveillance Study were followed‐up between July 2009 and February 2013. They were grouped into epilepsy‐ and non‐epilepsy‐related CSE, and compared with population norms and healthy controls using the Strengths and Difficulties Questionnaire; the Autism Spectrum Screening Questionnaire; and the Swanson, Nolan, and Pelham questionnaire. Children who scored above recommended clinical cut‐offs on any scale were invited for a neuropsychiatric assessment. Regression models were fitted to identify clinically relevant covariates associated with behavioural outcomes.

**Results:**

At a mean follow‐up of 8.1 years post‐CSE, 28% of enrolled children were found to have a psychiatric disorder. Children with epilepsy‐related CSE scored higher than norms on all scales and children with non‐epilepsy‐related CSE scored higher than norms on the Strengths and Difficulties Questionnaire and the Autism Spectrum Screening Questionnaire. Presence of seizures at baseline and recurrence of CSE was associated with worse outcomes in the group with epilepsy. Intellectual abilities were associated with behavioural outcomes in all participants.

**Interpretation:**

A large proportion of children manifest behavioural issues 8 years after CSE. The present data highlight the need for behavioural screening in children with neurodevelopmental impairments post‐CSE.

**What this paper adds:**

- Eight years post convulsive status epilepticus (CSE), 37% of parents report behavioural issues.
- Of enrolled children, 28% were found to have a Diagnostic and Statistical Manual mental disorder.
- Intellectual abilities are strongly associated with behavioural outcomes in children post‐CSE.

## What this paper adds

- Eight years post convulsive status epilepticus (CSE), 37% of parents report behavioural issues.
- Of enrolled children, 28% were found to have a Diagnostic and Statistical Manual mental disorder.
- Intellectual abilities are strongly associated with behavioural outcomes in children post‐CSE.

This article is commented on by Wright on pages https://doi.org/10.1111/dmcn.13700 of this issue.

This article's abstract has been translated into Spanish and Portuguese.

Follow the links from the http://onlinelibrary.wiley.com/doi/10.1111/dmcn.13636/abstract to view the translations.

## Resumen

**Objetivo:**

Describir el impacto en el comportamiento y en los trastornos psiquiátricos en niños, dentro de los 10 años después de presentar un estado epiléptico convulsivo (EEC).

**Metodo:**

Niños originalmente identificados por la encuesta poblacional del norte de Londres sobre vigilancia de la niñez con EEC fueron seguidos entre Julio del 2009 y Febrero del 2013. Ellos fueron agrupados en EEC relacionados con epilepsia y EEC no relacionado con epilepsia, y fueron comparados con normas de población y controles tipicos, usando el cuestionario de Fortalezas y Dificultades (Strengths and Difficulties Questionnaire), el Cuestionario tamizaje del espectro autista (Autism Spectrum Screening Questionnaire), y los cuestionarios de Swanson, Nolan, y Pelham (Swanson, Nolan, and Pelham questionnaire). Los que tuvieron un puntaje por encima de los valores límites recomendados en cualquier escala fueron invitados a realizar una evaluación neuropsiquiatrica. Se utilizaron modelos de regresión ajustados para identificar covariantes clínicamente relevantes asociadas con resultados del comportamiento.

**Resultados:**

Con una media de seguimiento de 8,1 años post EEC, 28% de los niños enrolados, tenían un trastorno psiquiátrico. Niños con EEC relacionado con epilepsia tuvieron puntajes más altos que la población con desarrollo típico en todas las escalas. Niños con EEC no relacionado a epilepsia tuvieron puntajes mayores que la población con desarrollo típico en el cuestionario de Fortalezas y Debilidades y en el Cuestionario tamizaje del espectro autista. La presencia de crisis epilépticas como base y la recurrencia de EEC fueron asociados a un peor resultado en el grupo de epilepsia. Habilidades intelectuales fueron asociadas con desarrollo conductual en todos los participantes.

**Interpretación:**

Una gran proporción de niños manifiestan trastornos del comportamiento 8 años después de EEC. El presente estudio recalca la importancia y la necesidad de pesquisa en niños con alteración del neurodesarrollo después de un EEC.

## Resumo

**Objetivo:**

Descrever o desfecho comportamental e psiquiátrico de crianças dentro de 10 anos de estado de mal epiléptico (EME).

**Métodos:**

Crianças originalmente identificadas por um estudo populacional no norte de Londres com EME em um estudo de vigilância da infância foram acompanhadas entre julho de 2009 e fevereiro de 2013. Eles foram agrupados em EME relacionado ou não relacionado a epilepsia e comparados com normas populacionais e controles saudáveis, utilizando O Questionário de pontos fortes e dificuldades (Strengths and Difficulties Questionnaire), Questionário de rastreio de espectro autista (Autism Spectrum Screening Questionnaire) e O Questionário de Swanson, Nolan e Pelham (Swanson, Nolan, and Pelham questionnaire). As crianças que pontuaram acima do ponto de corte clínico em qualquer escala foram convidados a realizar um avaliação neuropsiquiátrica. Modelos regressivos foram ajustados para identificar covariáveis clinicamente relevantes associadas aos resultados comportamentais.

**Resultados:**

Após um seguimento médio de 8,1 anos após EME, foi identificada desordem psiquiátrica em 28% das crianças participantes. Crianças com EME relacionado a epilepsia tiveram maiores pontuações do que as normas em todas as escalas e crianças com EME não relacionado a epilepsia pontuaram acima da norma no Questionário de pontos fortes e dificuldades e no Questionário de rastreio de espectro autista. A presença de crises convulsivas de base e recorrência do EME foram associados a piores desfechos no grupo com epilepsia. Habilidades intelectuais foram associadas ao desfecho comportamental em todos os participantes.

**Interpretaçâo:**

Uma grande proporção de crianças manifesta desordens comportamentais 8 anos após EME. Os presentes dados reforçam a necessidade de triagem comportamental em crianças com comprometimento do neurodesenvolvimento após EME.

**Abbreviations**
ASSQ: Autism Spectrum Screening Questionnaire
CSE: Convulsive status epilepticus
PFS: Prolonged febrile seizure
SDQ: Strengths and Difficulties Questionnaire
SNAP‐IV: Swanson, Nolan, and Pelham Questionnaire

Convulsive status epilepticus (CSE), the most common medical emergency in childhood,^1^ is associated with an increased risk of long‐term mortality,^2^ structural abnormalities in white matter and the hippocampal region,^3^, ^4^ neurocognitive and memory impairments,^5^, ^6^ and an overall worse quality of life.^7^ Aetiology is the major determinant of outcome.^8^ However, even children with no apparent pre‐existing neurological problems at CSE show evidence of short‐term structural and functional consequences after CSE.^9^, ^10^, ^11^, ^12^

Most data on long‐term outcomes of childhood CSE are collected from retrospective hospital‐based studies involving adults and children.^13^, ^14^ To our knowledge, there has only been one population‐based paediatric study investigating long‐term outcomes of childhood CSE, which focused only on neurological and intellectual outcomes.^15^ Our present study was specifically designed to determine behavioural outcomes of childhood CSE utilizing in‐depth standardized assessments and a clinical neuropsychiatric assessment. The inception cohort was from the first population‐based study focused on the epidemiology of childhood CSE, the North London Convulsive Status Epilepticus in Childhood Surveillance Study.^16^ Here we report our findings on the behavioural and psychiatric outcomes in this unique cohort within 10 years of the initial CSE episode.

## Method

### Participants and procedures

In this prospective cohort study, we aimed to recruit all surviving children after CSE originally identified during the North London Convulsive Status Epilepticus in Childhood Surveillance Study (detailed recruitment methods described elsewhere).^2^, ^16^ For each participant, demographic, medical, developmental data, neuropsychiatric diagnosis, and clinical details about their episode of CSE were obtained from the original North London Convulsive Status Epilepticus in Childhood Surveillance Study database and their hospital medical records. Subsequently, all participants and their parents were interviewed by one of the authors (SP) using a structured proforma to obtain information on the presence of seizures/epilepsy post‐CSE, CSE recurrence, behavioural problems, psychiatric diagnosis, schooling, and any drug treatment or interventions.

Recent studies point to high rates of psychiatric comorbidity in active epilepsy.^17^, ^18^, ^19^ We wanted to study the outcomes of children with CSE without the added burden of ongoing epileptic seizures and/or antiepileptic medication separately. Therefore, children were classified as (1) children who have received an epilepsy diagnosis at any time point pre‐ or post‐CSE (epilepsy‐related CSE) and (2) children who have never been diagnosed with epilepsy (non‐epilepsy‐related CSE).

For the recruitment of healthy controls, we sent emails to employees of Great Ormond Street Hospital and Young Epilepsy, a national epilepsy charity. Parents volunteered participation of their children. Five healthy patient siblings were also recruited. Exclusion criteria for healthy control participation were a diagnosis of epilepsy and/or the presence of neurodevelopmental delay, as reported by their parents. Indices of multiple deprivation were calculated for all participants (http://www.ons.gov.uk) and used as proxy measure for their socio‐economic status.

Study participants were sent questionnaires to be completed by their self‐nominated primary carer and invited for magnetic resonance imaging (MRI) and in‐person neuropsychological assessments at University College London Great Ormond Street Institute of Child Health/Great Ormond Street Hospital, London. Conventional MRI sequences were acquired on an Avanto 1.5 Tesla scanner (Siemens, Erlangen, Germany). Images were reviewed by two experienced paediatric neuroradiologists who agreed by consensus whether scans were (1) normal, (2) abnormal but of uncertain clinical significance, or (3) abnormal but clinically significant. The Wechsler Abbreviated Scale of Intelligence was used to obtain a Full‐scale IQ at this appointment.^20^

### Behavioural questionnaires

We selected three scales for parent completion that have been validated in large‐scale studies and are in widespread clinical use: (1) the Strengths and Difficulties Questionnaire (SDQ; a 25‐item checklist designed to screen for emotional, peer, conduct, and hyperactivity problems); (2) the Autism Spectrum Screening Questionnaire (ASSQ; a 27‐item checklist designed to screen for features of autism); and (3) the Swanson, Nolan, and Pelham Questionnaire (SNAP‐IV; an 18‐item checklist designed to screen for attention‐deficit–hyperactivity disorder [ADHD] traits). A higher score on these scales indicates the presence of more behavioural problems. The population mean for the SDQ (8.4 [SD 5.8]) was derived from the SDQ website (http://www.sdqinfo.org/). The ASSQ population mean (3.3 [SD 4.5]) was derived from the ‘Bergen Child Study’,^21^ and the SNAP‐IV population mean (0.75 [SD 0.75]) was derived from the study of Bussing et al.^22^ For scoring purposes we used the online SDQ algorithm that allowed us to extract an overall behaviour index and address missing items accordingly (https://sdqscore.org/Amber). The ASSQ and SNAP‐IV were scored manually. In instances of missing items we substituted those with the personal mean score, that is, average of remaining items on the scale. This method has been shown to be appropriate for dealing with missing data items (<2%).^23^ Those that passed an a priori determined clinical cut‐off (SDQ: 17; ASSQ: 17; SNAP‐IV: 1.67)^24^ on at least one behavioural questionnaire were invited along with their parents to attend a semi‐structured interview and assessment conducted by an experienced child and adolescent psychiatrist (CG).

### Neuropsychiatric assessment

Clinical diagnosis of psychiatric disorders was made by CG in accordance with the Diagnostic and Statistical Manual of Mental Disorders, 4th Edition criteria.^25^ All relevant material used in this semi‐structured interview can be found in Appendix S1 (online supporting information). In brief, the child and carers were interviewed using a set list of diagnostic question items and a neurological examination of the child was carried out in this session to detect the presence of developmental coordination disorder. At the beginning of the appointment, CG was blind to clinical details such as CSE group and results of previous investigations, but was aware that these children had scored higher than the clinical cut‐off on at least one screening questionnaire.

### Statistical analysis

Analyses were conducted with Predictive Analytics Software version 21 (IBM Chicago, IL, USA) for Windows. A *p* value of 0.050 was used as the cut‐off point for statistical significance. Independent sample *t*‐tests, analysis of variance, Mann–Whitney *U* tests, and *χ* ^2^ tests were used for group comparisons. One‐sample *t*‐test was conducted to compare group means with population‐derived means. We also ran the above analysis separately for patients with prolonged febrile seizures (PFS), who are known to be neurologically normal at CSE. A multivariate analysis of variance was performed to compare performance of the epilepsy CSE patient groups and controls on the behavioural scales. Bootstrapping using 1000 samples was applied to all our statistical tests as the distribution of some values was found to deviate from the normal distribution.

To reduce the dimensionality of our data set we ran a principal component analysis, which included all individual items on the SDQ (*n*=25), ASSQ (*n*=27), and SNAP‐IV (*n*=18) (total items=70). The principal component analysis resulted in a first principal component (mean=0, median=0.19), which accounted for the majority (84%) of variability in the behavioural outcomes data. For this reason, this single principal component (hereinafter behavioural outcomes factor) became the outcome of focus of the analyses. We ran univariable regression analyses separately for the two patient groups. The following independent covariates were investigated at the time of CSE: (1) age at CSE; (2) developmental delay reported by parents (yes/no); (3) prior seizure activity at baseline (yes/no); (4) duration of CSE (min); (5) whether CSE was continuous (yes/no); (6) whether CSE was focal (yes/no). We also included two clinical covariates collected at follow‐up: (7) recurrence of CSE (yes/no); (8) structural abnormalities present on the MRI as these have been shown to be associated with worse outcomes in other studies. Covariates associated with *p*<0.100 coefficients in the univariable analysis were entered into a linear regression to determine our final multivariable regression models for the groups.

### Ethics approval

The study was approved by the University College London Institute of Child Health/Great Ormond Street Hospital research ethics committee. We obtained written informed consent from all participants’ parents/guardians, and, where appropriate, we obtained assent from the participants.

## Results

**Figure 1:**
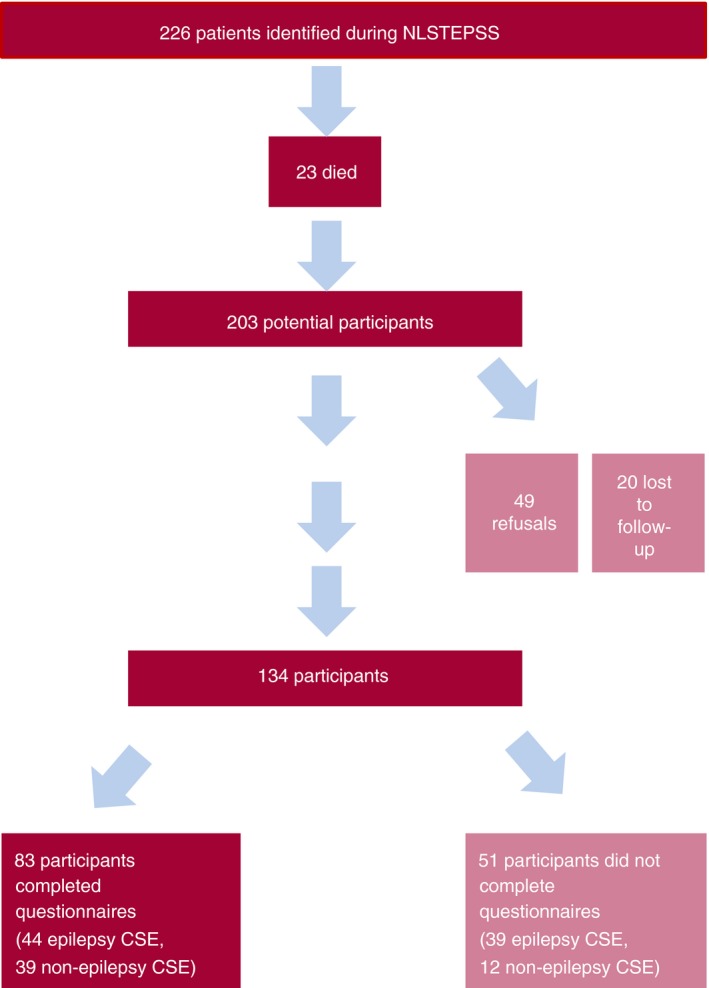
Patient recruitment flow chart. NLSTEPSS, North London Convulsive Status Epilepticus in Childhood Surveillance Study; CSE, convulsive status epilepticus. Non‐participants are indicated lighter shade. [Colour figure can be viewed at wileyonlinelibrary.com].

We had data available on 134 (66%) of 203 survivors from the inception CSE cohort, a mean of 8.1 years post‐CSE (range 5–10) (see Fig. 1 for recruitment details). There were no differences between the 134 study participants and the 69 dropouts on CSE related, clinical, and demographic characteristics (see Table 1).

**Table 1:** Demographic and clinical characteristics of participants with convulsive status epilepticus (CSE) and participants without (non‐CSE)

	CSE (*n*=134)	Non‐CSE (*n*=69)
Sex (female/male)	67/67	34/35
Mean (SD) age at CSE (mo)	46 (40.7)	50.3 (47)
Mean (SD) SES	33.2 (15)	35.3 (12.9)
Ethnicity		
White	54	20
Black	22	18
Asian	41	21
Mixed	7	4
Other	10	6
Full term (>36wks)	109/132 (83)	54/59 (92)
Seizures before CSE	77 (57.5)	40 (58)
Normal cognitive development before CSE^a^	68 (51)	37/63 (59)
Median (IQR) duration of CSE (min)	70 (45–100)	63 (50–101)
Focal CSE	48 (36)	38 (55)
Continuous CSE	70 (52)	29 (42)

^a^As reported by parents at the time of CSE. Data are *n* (%) unless otherwise indicated. SES, socio‐economic status; IQR, interquartile range.

Thirty‐one children (23%; 27 epilepsy CSE, four non‐epilepsy CSE) had a community neuropsychiatric diagnosis before entering the current study. With the exception of two children who had pre‐existing neuropsychiatric diagnosis at the time of their CSE, all children with CSE with neuropsychiatric diagnoses had their diagnosis during follow‐up. Nine were diagnosed as having ADHD and 22 as having autism spectrum disorder.

### Questionnaire results compared with population norms and controls

Of the 134 patients enrolled in our study, 83 provided completed behaviour questionnaires (see Table SI, online supporting information). *χ* ^2^ tests revealed that children who entered the study but did not complete behavioural questionnaires (*n*=51) were significantly more likely to be untestable on neuropsychological assessments owing to their cognitive impairments (*χ* ^2^ [2]=21.04; *p*<0.001) than the 83 who provided questionnaire data. Group comparisons between those who provided questionnaires (*n*=83) and dropouts (*n*=69) revealed no significant differences between the two groups.

Table 2 contains the means of scores for the SDQ, ASSQ, and SNAP‐IV for all groups. One‐sample *t*‐test revealed that (1) the epilepsy CSE group scored significantly higher than normative means on the SDQ (*p*=0.001), the ASSQ (*p*=0.001), and the SNAP‐IV (*p*=0.021) scales; (2) the non‐epilepsy CSE group scored significantly higher than the normative means on the SDQ (*p*=0.018) and the ASSQ (*p*=0.027) scales; and (3) the controls scored lower than the normative means on the SDQ (*p*=0.022) and the SNAP‐IV (*p*=0.001) scales. Additional *t*‐tests showed that children with PFS scored significantly higher than normative means on the ASSQ (*p*=0.026).

**Table 2:** Means for patient and control groups on the Strengths and Difficulties Questionnaire (SDQ); Autism Spectrum Screening Questionnaire (ASSQ); and Swanson, Nolan, and Pelham (SNAP) Questionnaire

Group	Epilepsy CSE	Non‐epilepsy CSE	Controls	Population‐derived norms
Sex (female/male)	20/24	19/20	9/7	NA
Age at test	13y 1mo^a^	10y 3mo^a^	12y 4mo^a^	NA
SES (SD)	33.6 (14.4)	31.6 (16.1)	26.9 (16.4)	NA
FSIQ (SD)	72.2 (17.6)^a^	100.9 (16.6)^a^	108.1 (13.2)^a^	100 (15)
SDQ (SD)	15.1 (7)	11 (6.7)^b^	5.3 (4.7)^b^	8.44 (5.8)
SDQ (range)	3–29	1–29	0–14	–
ASSQ (SD)	16.1 (11)	7.8 (8.6)	2.9 (3.8)	3.29 (4.5)
ASSQ (range)	0–40	0–39	0–15	
SNAP‐IV	1 (0.7)	0.5 (0.6)	0.3 (0.3)	0.75 (0.75)
SNAP‐IV (range)	0–2.4	0–2.6	0–1.1	–

^a^Statistically significant difference at the *p*<0.050 level. ^b^Statistically significant difference at the *p*<0.050 level on the multivariate analysis of variance with Full‐scale IQ (FSIQ) and age as covariates. CSE, convulsive status epilepticus; NA, not applicable.

On group comparisons, there were no differences in SES and sex between the two patient groups and controls. Non‐epilepsy children with CSE were younger at testing (*p*<0.001) and the epilepsy CSE group had lower Full‐scale IQ (*p*<0.001) in comparison with the control and the non‐epilepsy CSE groups. A multivariate analysis of variance with age at test and Full‐scale IQ as covariates and SDQ, ASSQ, and SNAP‐IV scores as dependent variables revealed a significant effect of Full‐scale IQ (*F* [3,74]=11.05; *p*<0.001) and a trend for an effect of group (*F* [3,75]=2.6; *p*=0.067) on behavioural scores. Pairwise comparisons revealed a trend for a difference between the non‐epilepsy CSE and the control group on the SDQ scale (*F* [2,76]=3.07; *p*=0.063).

### Regression analyses

Presence of seizures pre‐CSE (*F* [1,35]=8.23; *p*=0.008) and recurrent CSE [*F* (1,35)=2.21; *p*=0.075 (trend) were associated with the behavioural outcomes factor in the epilepsy CSE group in the univariable analyses (Table SII, online supporting information). Both (seizures: B=1.08 [*p*=0.008]; recurrence: B=0.64 [*p*=0.045]) were retained as significant covariates in our multivariable model of the behavioural outcomes factor (*R* ^2^=0.28; *p*=0.004). In the non‐epilepsy group, MRI abnormalities, focal CSE, and recurrence revealed trends for an association with the behavioural outcomes factor but were no longer significant covariates in the multivariable model.

### Neuropsychiatric assessment

**Figure 2:**
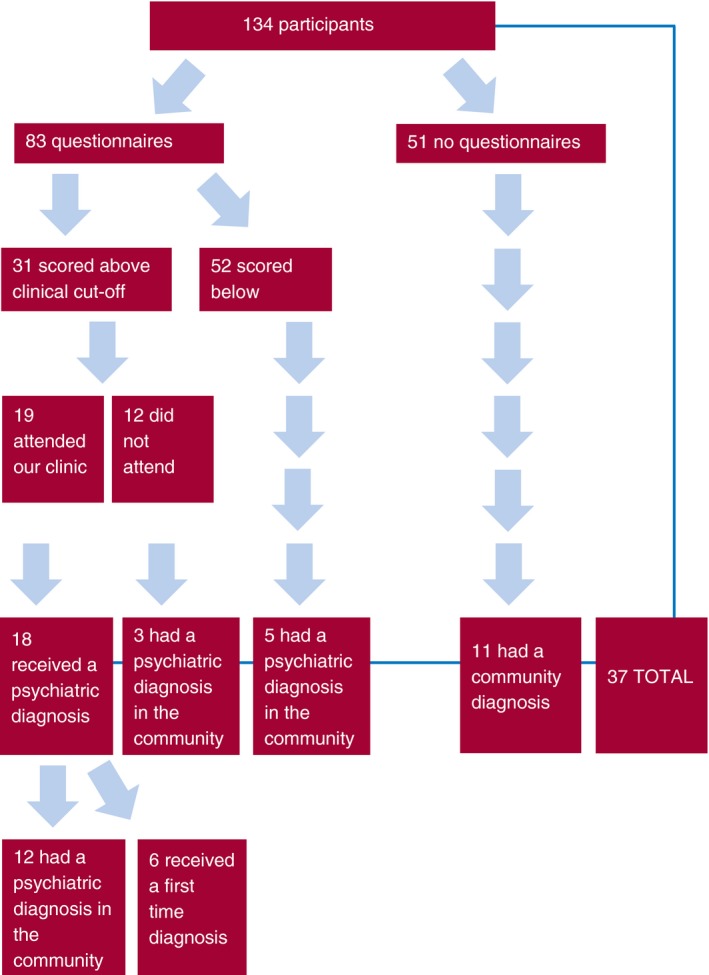
Psychiatric diagnosis flow chart. [Colour figure can be viewed at wileyonlinelibrary.com].

Thirty‐one patients (37% of respondents; 19 epilepsy CSE) were above the clinical cut‐off on at least one behavioural scale (Table SIII, online supporting information) and were invited for an in‐person neuropsychiatric assessment (see Fig. 2). Ten of the 29 children with PFS in our sample were also above at least one clinical cut‐off. Nineteen children (11 epilepsy CSE) had neuropsychiatric assessment. One family refused to attend and 11 families did not show up for their scheduled appointments. Three children out of the non‐attendees were already diagnosed with a psychiatric disorder (two with ADHD and one with autism spectrum disorder). Mann–Whitney *U* tests revealed no significant differences between children who did and did not attend the clinic appointment with the exception of the SNAP‐IV scale where those who attended had significantly higher SNAP‐IV scores (*p*=0.02) than non‐attendees.

All except one of the children who attended the clinic were diagnosed with a neuropsychiatric disorder (Table SIV, online supporting information). Six of these received a primary diagnosis of autism, eight received a primary diagnosis of ADHD, and three received a diagnosis of autism plus ADHD. One child was diagnosed as having pervasive developmental disorder not otherwise specified and four children met criteria for developmental coordination disorder. Six out of the 19 children were diagnosed with a disorder for the first time.

Of children who had a community diagnosis and a psychiatric assessment, there was concordance in diagnosis in 22. Of the remainder, an autism diagnosis in the community was revised to an ADHD diagnosis and an ADHD diagnosis was revised to an autism diagnosis. ADHD was codiagnosed in three children with autism, and developmental coordination disorder was codiagnosed in two children with autism and two children with ADHD.

In total, 37 of 134 enrolled participants (28%) had a psychiatric disorder in our cohort, many of whom had been diagnosed in the community. However, with additional assessment in this study, 15 of the 37 were either newly diagnosed conditions (*n*=6), had their diagnosis revised (*n*=2), or had an additional diagnosis (*n*=7). Of these, eight did not have epilepsy, so their behavioural problems cannot be attributed to ongoing epilepsy and/or the use of antiepileptic medications during a vulnerable developmental period.

## Discussion

This is the first population‐based study focused on long‐term behavioural outcomes after childhood CSE using carefully designed, standardized questionnaires, as well as in‐depth neuropsychiatric interviews. We found that 37% of patients, whose parents completed the questionnaires, revealed behavioural problems as they scored above the clinical cut‐off on at least one behavioural scale. In addition, 28% of patients had received a community diagnosis and/or were diagnosed with a DSM‐IV psychiatric disorder in the present study, including six children not previously identified in the community. The comparison of behavioural scores to sex‐ and socio‐economic status‐matched controls, as well as to population norms, confirmed the presence of behavioural issues in both the epilepsy and non‐epilepsy CSE groups.

A recent study published on quality of life in epilepsy found that CSE is independently associated with a worse quality of life.^7^ This may be directly related to the higher rate of behavioural issues that are reported in the present paper. Behavioural problems were found in 43% of children with epilepsy‐related CSE, which is similar to the prevalence of psychiatric disorders in children with epilepsy, regardless of a history of CSE.^17^, ^18^, ^19^ However, behavioural problems were still present in a high proportion of those with non‐epilepsy‐related CSE (31%), as well as children who had PFS (35%). Together these results could suggest that CSE itself, irrespective of aetiology, causes subsequent behavioural problems or that the effect of CSE on behaviour is mediated by another mechanism that predisposes the child to having both CSE and behavioural problems.

The lack of association between CSE seizure characteristics (e.g. duration) and behavioural outcomes in our study could suggest that it is not CSE per se that has a direct impact on outcome. However, all the children in the cohort had seizures lasting at least 30 minutes, so we cannot exclude the possibility of a lower critical threshold where seizure duration may affect behavioural outcomes. In addition, seizures before CSE, as well as CSE recurrence, were found to be associated with worse behavioural outcomes in the epilepsy CSE group. Other studies have also revealed a relationship between past seizure activity and worse outcomes,^4^ and such findings may be related to the multiple insult hypothesis, whereby CSE plus more seizures equals worse outcomes than CSE alone or seizures alone.

Our data show that children with behavioural difficulties may go undetected by medical services, possibly owing to a phenomenon known as ‘diagnostic overshadowing’ whereby the presence of another condition deprioritizes the detection of other conditions. Conversely, it is equally plausible that children that have no ongoing medical issues do not get seen by medical professionals and their problems go undetected for years. This seems to be true of the children with non‐epilepsy CSE as only four were diagnosed with a psychiatric condition in the community, whereas 12 scored higher than clinical cut‐offs in our study, and eight of 39 respondents were diagnosed with a DSM‐IV disorder. Full‐scale IQ was shown to have a strong association with long‐term behavioural outcomes, which suggests that it could be useful in the early identification of children with behavioural problems post‐CSE.

### Limitations

A possible limitation of the present study is that only 41% of the inception cohort returned our questionnaire. However, the baseline characteristics of children who provided completed questionnaires and dropouts were well matched. Non‐completion of questionnaires in the enrolled sample was elected by parents whose children were more likely to be neurologically compromised at baseline; diagnosed with epilepsy during follow‐up; and unable to complete neuropsychological assessments. While our questionnaires were selected to pick up a wide gamut of behavioural issues, many of the composing items are not applicable in children with severe intellectual disability. The fact that parents of children with more neurodevelopmental problems opted out of completing our questionnaires combined with the finding that intellectual abilities are tightly linked to mental health suggests that our estimates of behavioural issues in children after CSE are most likely conservative. An in‐person assessment for each study participant would have been ideal, although more resource intensive. Likewise, standardized neuropsychological testing at CSE baseline would have been preferable to parental/medical report of abilities,^6^ utilized as a proxy of developmental delay in this study. Such formal measures would have allowed the recruitment of control groups equated for Full‐scale IQ at baseline and led to the systematic apportioning of CSE and other contributions to long‐term behavioural outcomes.

In addition, it could be argued that it would have been more appropriate to classify CSE into PFS and non‐PFS, but even when children with PFS were looked at in isolation they still showed significant behavioural problems, which is consistent with the report of developmental problems in children with PFS at 1‐year post‐CSE.^6^, ^12^ Finally, adding a control group with motor and cognitive impairments unrelated to a diagnosed neurological condition may have been helpful in teasing apart the contributions to behavioural outcomes of CSE/epilepsy‐related and motor/cognitive‐related factors, but such a group would have been difficult to obtain.

## Conclusion

A large proportion of children manifest behavioural and psychiatric issues 8 years post‐CSE. These findings support the systematic screening of children for behavioural/psychiatric disorders post‐CSE akin to the ones proposed for new‐onset epilepsies.^26^ Intellectual abilities have been shown to be strongly correlated with behavioural outcomes and can therefore be used in guiding the early identification of vulnerable cases.

## Supporting information


**Appendix S1:** All questionnaires and diagnostic materials used in the neuropsychiatric interview.

**Table SI:** Demographic and clinical characteristics of participants with questionnaires, participants without questionnaires, and non‐participants
**Table SII:** Behavioural outcomes factor univariable regression results
**Table SIII:** Number of participants scoring above clinical cut‐offs on Strengths and Difficulties; Autism Spectrum Screening; and Swanson; Nolan, and Pelham questionnaires
**Table IV:** Diagnosis for children who attended neuropsychiatric interview
