# Supplementary material for: Long‐term behavioural outcomes after paediatric convulsive status epilepticus: a population‐based cohort study
Source: Dev Med Child Neurol. 2017 Dec 10;60(4):409–16. doi: 10.1111/dmcn.13636 (PMC5900729; doi:10.1111/dmcn.13636)
Supplement: Supplementary file 1 — Appendix S1: All questionnaires and diagnostic materials used in the neuropsychiatric interview. [file DMCN-60-409-s002.docx]

Appendix 1: All questionnaires and diagnostic materials used in the neuropsychiatric interview

**DSM-V Autism Spectrum Disorder**

Must meet criteria A, B, C, and D:

A.    Persistent deficits in social communication and social interaction across contexts, not accounted for by general developmental delays, and manifest by all 3 of the following:

1.     Deficits in social-emotional reciprocity; ranging from abnormal social approach and failure of normal back and forth conversation through reduced sharing of interests, emotions, and affect and response to total lack of initiation of social interaction,

2.     Deficits in nonverbal communicative behaviors used for social interaction; ranging from poorly integrated- verbal and nonverbal communication, through abnormalities in eye contact and body-language, or deficits in understanding and use of nonverbal communication, to total lack of facial expression or gestures.

3.     Deficits in developing and maintaining relationships, appropriate to developmental level (beyond those with caregivers); ranging from difficulties adjusting behavior to suit different social contexts through difficulties in sharing imaginative play and  in making friends  to an apparent absence of interest in people

B.    Restricted, repetitive patterns of behavior, interests, or activities as manifested by at least two of  the following:

1.     Stereotyped or repetitive speech, motor movements, or use of objects; (such as simple motor stereotypies, echolalia, repetitive use of objects, or idiosyncratic phrases).

2.     Excessive adherence to routines, ritualized patterns of verbal or nonverbal behavior, or excessive resistance to change; (such as motoric rituals, insistence on same route or food, repetitive questioning or extreme distress at small changes).

3.     Highly restricted, fixated interests that are abnormal in intensity or focus; (such as strong attachment to or preoccupation with unusual objects, excessively circumscribed or perseverative interests).

4.     Hyper-or hypo-reactivity to sensory input or unusual interest in sensory aspects of environment; (such as apparent indifference to pain/heat/cold, adverse response to specific sounds or textures, excessive smelling or touching of objects, fascination with lights or spinning objects).

C.    Symptoms must be present in early childhood (but may not become fully manifest until social demands exceed limited capacities)

D.    Symptoms together limit and impair everyday functioning.

**DSM IV CRITERIA FOR DIAGNOSING AUTISTIC DISORDER***

**A. A total of six (or more) items from (1), (2), and (3), with at least two from (1), and one each from (2) and (3):**

1. Qualitative impairment in social interaction, as manifested by at least two of the following:

a. marked impairment in the use of multiple nonverbal behaviors such as eye-to-eye gaze, facial expression, body postures, and gestures to regulate social interaction.

b. failure to develop peer relationships appropriate to developmental level.

c. a lack of spontaneous seeking to share enjoyment, interests or achievements with other people (e.g., by a lack of showing, bringing or pointing out objects of interest.

d. lack of social or emotional reciprocity.

2. Qualitative impairments in communication as manifested by at least one of the following:

a. delay in, or total lack of, the development of spoken language (not accompanied by an attempt to compensate through alternative modes of communication such as gesture or mime).

b. in individuals with adequate speech, marked impairment in the ability to initiate or sustain a conversation with others.

c. stereotyped and repetitive use of language or idiosyncratic language.

d. lack of varied, spontaneous, make-believe play or social imitative play appropriate to developmental level.

3. Restricted, repetitive and stereotyped patterns of behavior, interests and activities, as manifested by at least one of the following:

a. encompassing preoccupation with one or more stereotyped and restricted patterns of interest that is abnormal either in intensity or focus.

b. apparently inflexible adherence to specific nonfunctional routines or rituals.

c. stereotyped and repetitive motor mannerisms (e.g., hand or finger flapping or twisting, or complex whole-body movements).

d. persistent preoccupation with parts of objects.

**B. Delays or abnormal functioning in at least one of the following areas, with onset prior to age 3 years: (1) social interaction, (2) language as used in social communication, or (3) symbolic or imaginative play.**

**C. The disturbance is not better accounted for by Rett's Disorder or Childhood Disintegrative Disorder.**

**Reference**

*American Psychiatric Association. (2000). *Diagnostic and statistical manual of mental disorders* (4th ed., rev.). Washington DC: Author.

(Pg. 75)


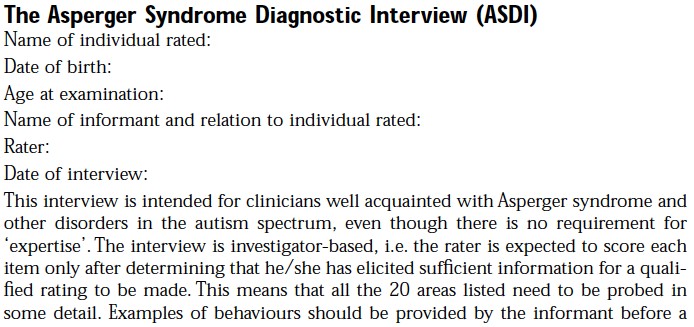

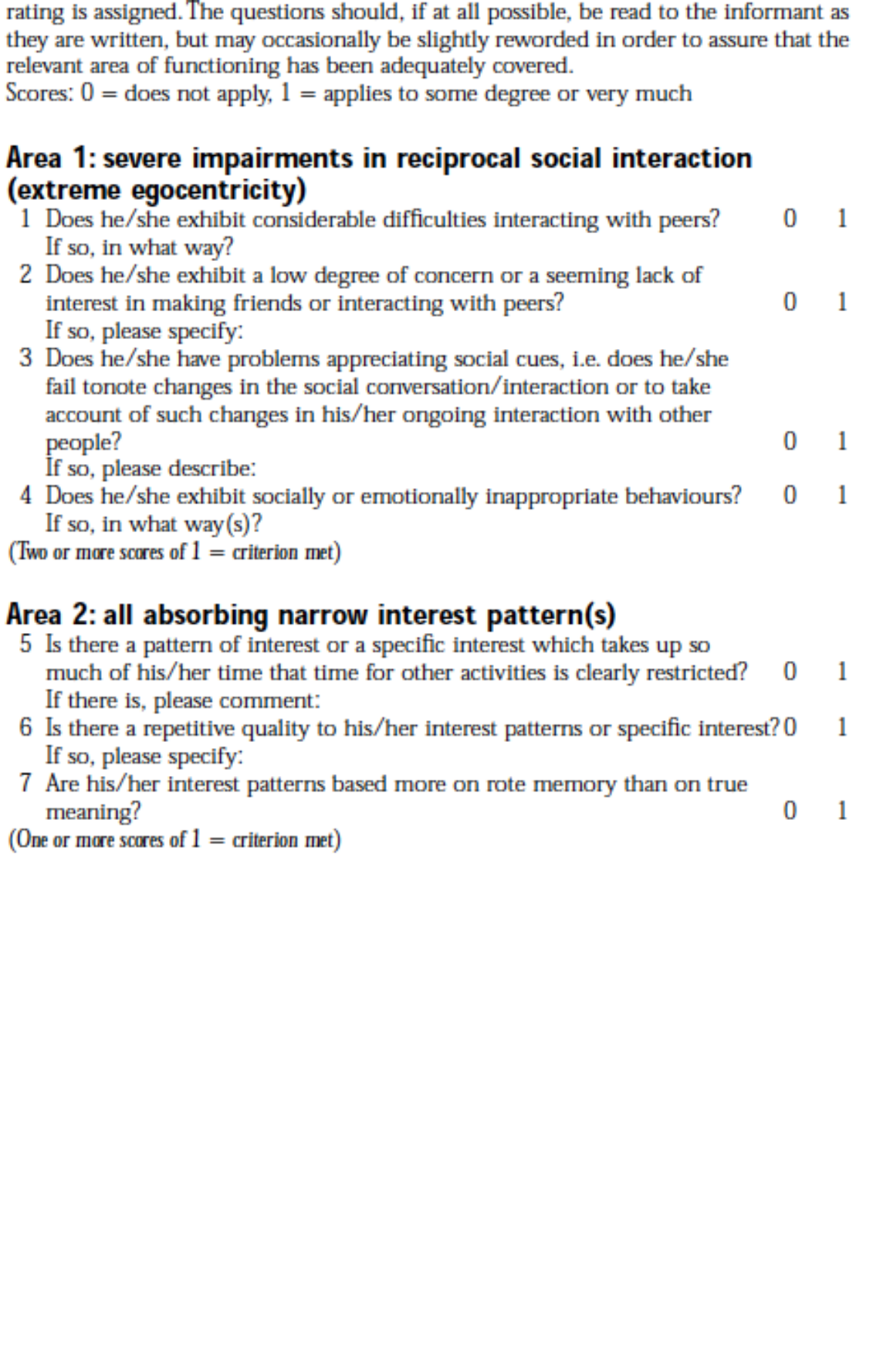

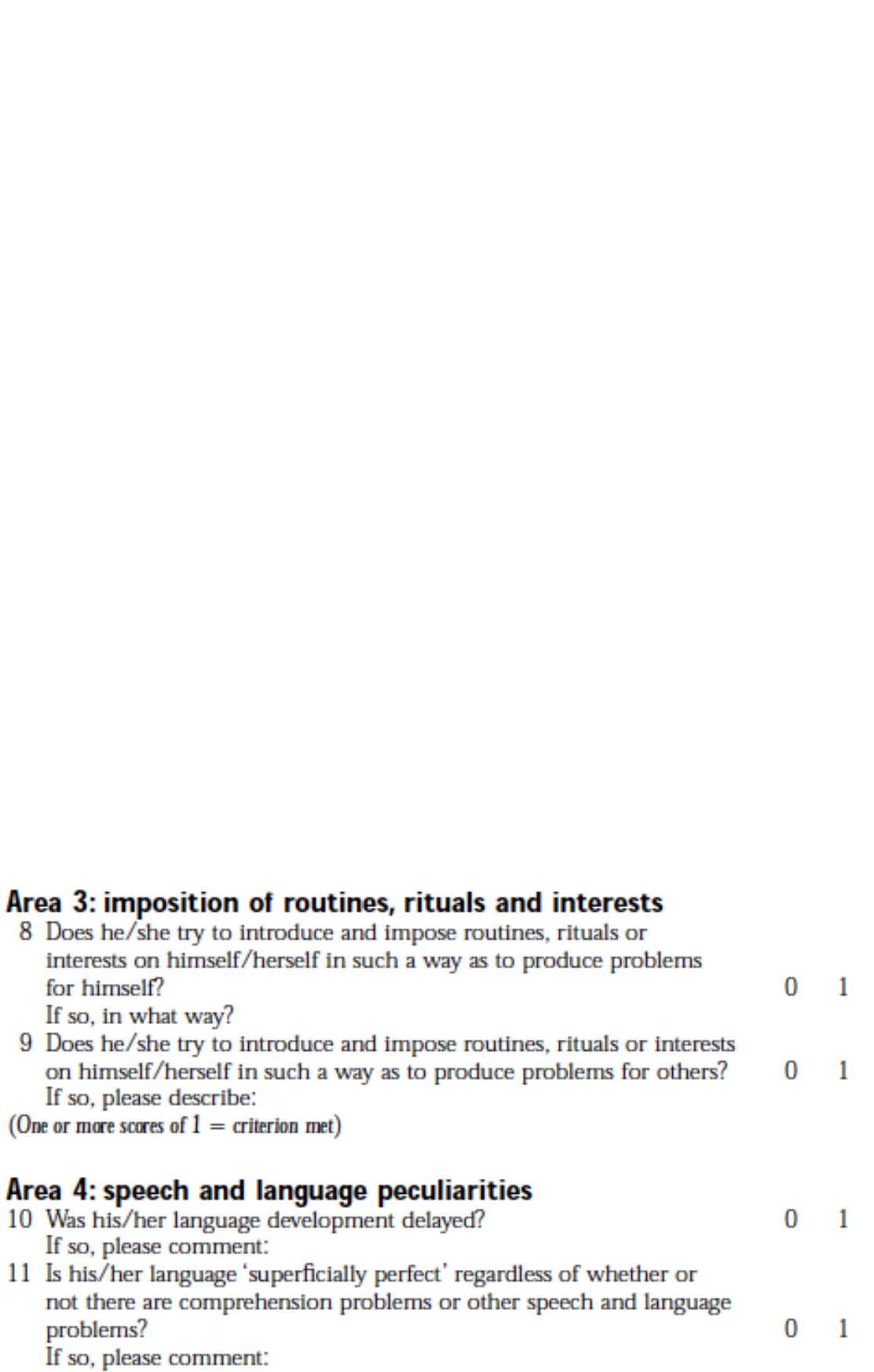

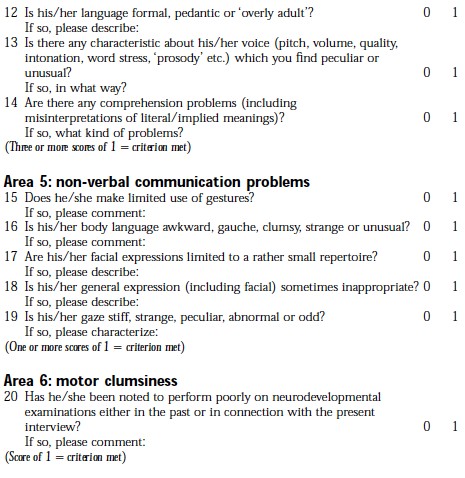


**DSM-V Diagnostic Criteria for Attention Deficit/Hyperactivity Disorder**

A. Either (1) and/or (2).

1. **Inattention:** Six (or more) of the following symptoms have persisted for at least 6 months to a degree that is inconsistent with developmental level and that impact directly on social and academic/occupational activities. ***Note:*** for older adolescents and adults (ages 17 and older), only 4 symptoms are required. The symptoms are not due to oppositional behavior, defiance, hostility, or a failure to understand tasks or instructions.

(a) Often ***fails to give close attention to details*** or makes careless mistakes in schoolwork, at work, or during other activities (for example, overlooks or misses details, work is inaccurate).

(b) Often has ***difficulty sustaining attention*** in tasks or play activities (for example, has difficulty remaining focused during lectures, conversations, or reading lengthy writings).

(c) Often ***does not seem to listen*** when spoken to directly (mind seems elsewhere, even in the absence of any obvious distraction).

(d) Frequently ***does not follow through*** on instructions (starts tasks but quickly loses focus and is easily sidetracked, fails to finish schoolwork, household chores, or tasks in the workplace).

(e) Often has ***difficulty organizing tasks*** and activities. (Has difficulty managing sequential tasks and keeping materials and belongings in order. Work is messy and disorganized. Has poor time management and tends to fail to meet deadlines.)

(f) Characteristically avoids, seems to dislike, and is ***reluctant to engage in tasks that require sustained mental effort*** (such as schoolwork or homework or, for older adolescents and adults, preparing reports, completing forms, or reviewing lengthy papers).

(g) Frequently ***loses objects*** necessary for tasks or activities (e.g., school assignments, pencils, books, tools, wallets, keys, paperwork, eyeglasses, or mobile telephones).

(h) Is often ***easily distracted*** by extraneous stimuli. (for older adolescents and adults may include unrelated thoughts.).

(i) Is often ***forgetful*** in daily activities, chores, and running errands (for older adolescents and adults, returning calls, paying bills, and keeping appointments).

2. **Hyperactivity and Impulsivity:** Six (or more) of the following symptoms have persisted for at least 6 months to a degree that is inconsistent with developmental level and that impact directly on social and academic/occupational activities. ***Note:*** for older adolescents and adults (ages 17 and older), only 4 symptoms are required. The symptoms are not due to oppositional behavior, defiance, hostility, or a failure to understand tasks or instructions.

(a) Often ***fidgets*** or taps hands or feet or squirms in seat.

(b) Is often ***restless*** during activities when others are seated (may leave his or her place in the classroom, office or other workplace, or in other situations that require remaining seated).

(c) Often ***runs about*** or climbs on furniture and moves excessively in inappropriate situations. In adolescents or adults, may be limited to feeling restless or confined.

(d) Is often ***excessively loud*** or noisy during play, leisure, or social activities.

(e) Is often ***“on the go,”*** acting as if “driven by a motor.” Is uncomfortable being still for an extended time, as in restaurants, meetings, etc. Seen by others as being restless and difficult to keep up with.

(f) Often ***talks excessively***.

(g) Often ***blurts out an answer*** before a question has been completed. Older adolescents or adults may complete people’s sentences and “jump the gun” in conversations.

(h) Has ***difficulty waiting his or her turn*** or waiting in line.

(i) Often ***interrupts or intrudes*** on others (frequently butts into conversations, games, or activities; may start using other people’s things without asking or receiving permission, adolescents or adults may intrude into or take over what others are doing).

(j) Tends to ***act without thinking***, such as starting tasks without adequate preparation or avoiding reading or listening to instructions. May speak out without considering consequences or make important decisions on the spur of the moment, such as impulsively buying items, suddenly quitting a job, or breaking up with a friend.

(k) Is often ***impatient***, as shown by feeling restless when waiting for others and wanting to move faster than others, wanting people to get to the point, speeding while driving, and cutting into traffic to go faster than others.

(l) Is ***uncomfortable doing things slowly and systematically*** and often rushes through activities or tasks.

(m) Finds it ***difficult to resist temptations or opportunities***, even if it means taking risks (A child may grab toys off a store shelf or play with dangerous objects; adults may commit to a relationship after only a brief acquaintance or take a job or enter into a business arrangement without doing due diligence).

B. Several noticeable inattentive or hyperactive-impulsive symptoms were present by age 12.

C. The symptoms are apparent in two or more settings (e.g., at home, school or work, with friends or relatives, or in other activities).

D. There must be clear evidence that the symptoms interfere with or reduce the quality of social, academic, or occupational functioning.

E. The symptoms do not occur exclusively during the course of schizophrenia or another psychotic disorder and are not better accounted for by another mental disorder (e.g., mood disorder, anxiety disorder, dissociative disorder, or a personality disorder).

***Specify Based on Current Presentation***

**Combined Presentation:** If both Criterion A1 (Inattention) and Criterion A2 (Hyperactivity-Impulsivity) are met for the past 6 months.

**Predominately Inattentive Presentation:** If Criterion A1 (Inattention) is met but Criterion A2 (Hyperactivity-Impulsivity) is not met *and* 3 or more symptoms from Criterion A2 have been present for the past 6 months.

**Predominately Hyperactive/Impulsive Presentation:** If Criterion A2 (Hyperactivity- Impulsivity) is met and Criterion A1 (Inattention) is not met for the past 6 months.

**Inattentive Presentation (Restrictive):** If Criterion A1 (Inattention) is met but no more than 2 symptoms from Criterion A2 (Hyperactivity-Impulsivity) have been present for the past 6 months.!

**Disorders Usually First Diagnosed In Infancy, Childhood, or Adolescence**

**Mental Retardation**

317 Mild mental retardation

318.0 Moderate mental retardation

318.1 Severe mental retardation free burress

318.2 Profound mental retardation

319 Mental retardation, severity unspecified

**Learning disorders**

315.00 Reading disorder

315.1 Mathematics disorder

315.2 Disorder of written expression

315.9 Learning disorder NOS

**Motor skills disorders**

315.4 Developmental coordination disorder

**Communication disorders**

315.31 Expressive language disorder

315.32 Mixed receptive-expressive language disorder

315.39 Phonological disorder

307.0 Stuttering

307.9 Communication disorder NOS

**Pervasive developmental disorders**

299.00 Autistic Disorder

299.80 Rett's Disorder

299.10 Childhood Disintegrative Disorder

299.80 Asperger’s Disorder

299.80 Pervasive Developmental Disorder NOS

**Attention-deficit and disruptive behavior disorders**

▪ Attention-Deficit Hyperactivity Disorder

▪ 314.01 Combined subtype

▪ 314.01 Predominantly hyperactive-impulsive subtype

▪ 314.00 Predominantly inattentive subtype

▪ 314.9 Attention-Deficit Hyperactivity Disorder NOS

▪ Conduct disorder

▪ 312.81 Childhood onset

▪ 312.82 Adolescent onset

▪ 312.89 Unspecified onset

▪ 313.81 Oppositional Defiant Disorder

▪ 312.9 Disruptive Behavior Disorder NOS

**Feeding and eating disorders of infancy or early childhood**

307.52 Pica

307.59 Feeding disorder of infancy or early childhood

**Tic disorders**

307.23 Tourette’s Disorder

307.22 Chronic motor or vocal tic disorder

307.21 Transient tic disorder

307.20 Tic disorder NOS

**Elimination disorders**

▪ Encopresis

▪ 787.6 Encopresis, with constipation and overflow incontinence

▪ 307.7 Encopresis, without constipation and overflow incontinence

▪ 307.6 Enuresis (not due to a general medical condition)

**Other disorders of infancy, childhood, or adolescence**

309.21 Separation anxiety disorder

313.23 Selective mutism

313.89 Reactive attachment disorder of infancy or early childhood

307.3 Stereotypic movement disorder

313.9 Disorder of infancy, childhood, or adolescence NOS

ADHD Rating Scale

Child’s Name:

Age:

Date:

Completed By:

Parent

Teacher

Other

For each line below, please put an “x” in the box that best describes the child's behaviour over the last 6 months

|  | BEHAVIOUR | Always or very often | Often | Somewhat | Rarely or  Never |
| --- | --- | --- | --- | --- | --- |
| Inattention | Fails to give close attention to details or makes careless mistakes in schoolwork/homework. |  |  |  |  |
|  | Has difficulty keeping attention on tasks or play activities. |  |  |  |  |
|  | Does not seem to listen when spoken to directly. |  |  |  |  |
|  | Does not follow through on instructions and fails to finish schoolwork or chores. |  |  |  |  |
|  | Has difficulty organizing tasks and activities. |  |  |  |  |
|  | Avoids or strongly dislikes tasks that require sustained mental effort (e.g., homework) |  |  |  |  |
|  | Loses things necessary for tasks or activities (e.g., pencils, books, toys, etc). |  |  |  |  |
|  | Is easily distracted by outside stimuli. |  |  |  |  |
|  | Is forgetful in daily activities. |  |  |  |  |
|  | TOTALS for Inattention |  |  |  |  |
| Impulsivity and Hyperactivity | Fidgets with hands or feet or squirms in seat. |  |  |  |  |
|  | Leaves seat in situations in which remaining seated is expected (e.g., dinner table). |  |  |  |  |
|  | Runs about or climbs in situations where it is inappropriate. |  |  |  |  |
|  | Has difficulty playing quietly. |  |  |  |  |
|  | Is “on the go” or acts “driven by a motor.” |  |  |  |  |
|  | Talks excessively. |  |  |  |  |
|  | Blurts out answers to questions before the questions have been completed. |  |  |  |  |
|  | Has difficulty awaiting turn. |  |  |  |  |
|  | Interrupts others or intrudes on others (e.g., butts into games) |  |  |  |  |
|  | TOTALS for Hyperactivity and Impulsivity |  |  |  |  |

Were some of these behaviours present before age 7? Yes

No

Unsure

N/A

| Children’s Global Assessment Scale (CGAS)  David Shaffer, M.D., Madelyn S. Gould, Ph.D. Hector Bird, M.D., Prudence Fisher, B.A. Adaptation of the Adult Global Assessment Scale (Robert L. Spitzer, M.D., Nathan Gibbon, M.S.W., Jean Endicott, Ph.D.)  **PLEASE RECORD A CGAS SCORE EVEN IF THIS IS BASED ON YOUR MEMORY OF THE YOUNG PERSON’S FUNCTIONING AT THE TIME OF REFERRAL. THE DATE OF RATING IS REQUIRED ONLY IF THIS WAS RECORDED CLOSE TO THE TIME OF THE**  **‘INDEX’ REFERRAL.** | |
| --- | --- |
| **43a** | **DATE OF CGAS RATING:** ……../……../……..  **OR FROM MEMORY (PLEASE INDICATE AS APPROPRIATE)**  **(IF RECORDED CLOSE TO TIME OF ‘INDEX’ REFERRAL)** |
| **100-91** | **DOING VERY WELL**  Superior functioning in all areas (at home, at school and with peers), involved in a range of activities and has many interests (e.g. has hobbies or participates in extracurricular activities or belongs to an organised group such as Scouts, etc.). Likeable, confident, everyday worries never get out of hand. Doing well in school. No symptoms. |
| **90 – 81** | **DOING WELL**  Good functioning in all areas. Secure in family, school, and with peers. There may be transient difficulties and "everyday" worries that occasionally get out of hand (e.g. mild anxiety associated with an important exam, occasionally "blow-ups" with siblings, parents or peers). |
| **80 – 71** | **DOING ALL RIGHT –minor impairment**  No more than slight impairment in functioning at home, at school or with peers. Some disturbance of behaviour or emotional distress may be present in response to life stresses (e.g. parental separations, deaths, birth of a sibling) but these are brief and interference with functioning is transient; such children are only minimally disturbing to others and are not considered  deviant by those who know them. |
| **70 – 61** | **SOME PROBLEMS - in one area only**  Some difficulty in a single area, but generally functioning pretty well, (e.g. sporadic or isolated antisocial acts such as occasionally playing hooky, petty theft; consistent minor difficulties with school work, mood changes of brief duration, fears and anxieties which do not lead to gross avoidance behaviour; self-doubts). Has some meaningful interpersonal relationships. Most people who do not know the child well would not consider him/her deviant but those who do know him/her well might express concern. |
| **60 – 51** | **SOME NOTICEABLE PROBLEMS – in more than one area**  Variable functioning with sporadic difficulties or symptoms in several but not all social areas. Disturbance would be apparent to those who encounter the child in a dysfunctional setting or time but not to those who see the child in other settings. |
| **50 – 41** | **OBVIOUS PROBLEMS – moderate impairment in most areas or severe in one area**  Moderate degree of interference in functioning in most social areas or severe impairment functioning in one area, such as might result from, for example, suicidal preoccupations and ruminations, school refusal and other forms of anxiety, obsessive rituals, major conversion symptoms, frequent anxiety attacks, frequent episodes of aggressive or other antisocial behaviour with some preservation of meaningful social relationships. |
| **40 – 31** | **SERIOUS PROBLEMS – major impairment in several areas and unable to function in one area**  Major impairment in functioning in several areas and unable to function in one of these areas, i.e. disturbed at home, at school, with peers or in the society at large, e.g. persistent aggression without clear instigation; markedly withdrawn and isolated behaviour due to either mood or through disturbance, suicidal attempts with clear lethal intent. Such children are likely to require special schooling and/or hospitalisation or withdrawal from school (but this is not a sufficient criterion for inclusion in this category). |
| **30 – 21** | **SEVERE PROBLEMS - unable to function in almost all situations**  Unable to function in almost all areas, e.g. stays at home, in ward or in bed all day without taking part in social activities OR  severe impairment in reality testing OR serious impairment in communication (e.g. sometimes incoherent or inappropriate). |
| **20 – 11** | **VERY SEVERELY IMPAIRED -considerable supervision is required for safety**  Needs considerable supervision to prevent hurting others or self, e.g. frequently violent, repeated suicide attempts OR to maintain personal hygiene! OR gross impairment in all forms of communication, e.g. severe abnormalities in verbal and gestural communication, marked social aloofness, stupor, etc. |
| **10 – 1** | **EXTREMELY IMPAIRED - constant supervision is required for safety**  Needs constant supervision (24-hour care) due to severely aggressive or self-destructive behaviour or gross impairment in reality testing, communication, cognition, affect or personal hygiene. |
|  | Specified time period: 1 month |
| **43b** | **CGAS SCORE =** |
