# Supplementary material for: Long‐term behavioural outcomes after paediatric convulsive status epilepticus: a population‐based cohort study
Source: Dev Med Child Neurol. 2017 Dec 10;60(4):409–16. doi: 10.1111/dmcn.13636 (PMC5900729; doi:10.1111/dmcn.13636)
Supplement: Supplementary file 2 — Table SI: Demographic and clinical characteristics of participants with questionnaires, participants without questionnaires, and non‐participants Table SII: Behavioural outcomes factor univariable regression results Table SIII: Number of participants scoring above clinical cut‐offs on Strengths and Difficulties; Autism Spectrum Screening; and Swanson; Nolan, and Pelham questionnaires Table IV: Diagnosis for children who attended neuropsychiatric interview [file DMCN-60-409-s001.doc]

**Supplemental Table 1 Demographic and clinical characteristics of participants with questionnaires, participants without questionnaires and non-participants**

|  | **Participants with questionnaires**  **(N=83)** | **Participants with no questionnaires**  **(N=51)** | **Non-participants (N=69)** |
| --- | --- | --- | --- |
| **Gender (Female: Male)** | 39:44 | 28:23 | 34:35 |
| **Age at CSE in Months (SD)** | 40.6 (37.4) | 55.3 (44.5) | 50.3 (47) |
| **Age at follow-up** | **11 years 7 months^&^** | **13 years 4 months^&^** | n/a |
| **SES (SD)** | 32.3 (15.3) | 34.6 (14.5) | 35.3 (12.9) |
| **Ethnic Category** | 35 white  13 black  24 Asian  5 mixed  6 other | 19 white  9 black  17 Asian  2mixed  4 other | 20 white  18 black  21 Asian  4 mixed  6 other |
| **Full term (>36 weeks)** | 67/81 (82.7%) | 42 (82.4%) | 54/59 (78.3%) |
| **Epilepsy** | **44 (53%)*** | **39 (76.5%)*** | n/a |
| **Normal MRI at follow-up** | 38/65(58.5%) | 10/20 (50%) | n/a |
| **Seizures prior to CSE** | 46(55.4%) | 32 (62.7%) | 40 (58%) |
| **Cognitive delays prior to CSE^#^** | **31/81 (38.3%)*** | **32 (62.7%)*** | **26/63 (41.2%)*** |
| **Duration of CSE in minutes (SD)** | 84.80 (82.9) | 109.2 (142.3) | 78.4 (39.7) |
| **Focal CSE (%)** | 30 (36.1%) | 33 (64.7%) | 38 (55.1%) |
| **Continuous CSE (%)** | 44 (53%) | 26 (51%) | 29 (42%) |
| **Untestable on FSIQ** | **11 (13.5%)*** | **25 (49%)*** | n/a |

*Significant difference at p<0.05 using a Chi square test.

^&^Significant difference at p<0.05 using an independent samples t-test.

#as reported by parents at the time of CSE

**Abbreviations:** Convulsive status epilepticus (CSE), full scale intelligence quotient (FSIQ), socioeconomic status (SES), number (N), standard deviation (SD), not available (n/a).

**Supplemental Table 2 BOF univariable regression results**

|  | **Epilepsy CSE** | **non-epilepsy CSE** |
| --- | --- | --- |
| **Age at CSE in Months** | B=-0.003, p=0.44 | B=0.15, p=0.16 |
| **MRI visible abnormalities** | B=0.22, p=0.24 | B=-0.25, p=0.09 |
| **Seizures prior to CSE** | **B=1.01, p=0.008** | B=-0.10, p=0.76 |
| **Cognitive delays at CSE** | B=0.44, p=0.22 | B=0.85, p=0.32 |
| **Duration of CSE** | B=-0.002, p=0.49 | B=0.001, p=0.24 |
| **Focal CSE** | B=0.12, p=0.68 | B=-0.45, p=0.08 |
| **Continuous CSE** | B=-0.12, p=0.72 | B=0.48, p=0.11 |
| **CSE recurrence** | B=0.53, p=0.07 | B=-0.53, p=0.09 |

**Abbreviations:** Convulsive status epilepticus (CSE), magnetic resonance imaging (MRI)

**Supplemental Table 3 Number of participant scoring above** clinical cut-offs on SDQ, ASSQ and SNAP IV questionnaires

| Group | Epilepsy CSE | Non-epilepsy CSE |
| --- | --- | --- |
| SDQ | 15 (35.7%) | 10 (25.6%) |
| ASSQ | 14 (38.9%) | 5 (13.9%) |
| SNAP | 8 (21.1%) | 2 (5.7%) |

Number of participants that failed clinical cut-offs on the SDQ (≥17), ASSQ (≥17), and ADHD (≥1.67) scales. Percentages in brackets represent the percentage of children that failed these cut-offs. **Abbreviations:** Convulsive Status Epilepticus (CSE), Strengths and Difficulties questionnaire (SDQ), Autism Spectrum Screening questionnaire (ASSQ), Swanson, Nolan, and Pelham questionnaire (SNAP), number of participants per scale (n).

**Supplemental Table 4** Diagnosis for children who attended neuropsychiatric interview

| Patient | Age | Gender | Cause | Epilepsy | FSIQ | SDQ | ASSQ | SNAP-IV | ASD | ADHD | PDD-NOS | DCD |
| --- | --- | --- | --- | --- | --- | --- | --- | --- | --- | --- | --- | --- |
| 1 | 9 | F | PFS | NO | 98 | 12 | **18** | N/A |  | + |  | + |
| 2 | 8 | M | PFS | NO | 114 | **18** | 10 | .9 |  | + |  |  |
| 3 | 9 | F | PFS | NO | 92 | **23** | **19** | 1.1 |  | + |  |  |
| 4 | 7 | M | PFS | NO | 100 | **25** | 10 | 1.2 |  |  |  |  |
| 5 | 11 | M | PFS | NO | 90 | **18** | 8 | 1.4 |  |  | + |  |
| 6 | 12 | M | PFS | NO | 84 | **17** | 2 | .8 |  | + |  |  |
| 7 | 14 | M | UNC | NO | 57 | **29** | **39** | **2.6** | + |  |  | + |
| 8 | 10 | M | PFS | YES | 74 | 16 | **30** | 1.6 | + |  |  |  |
| 9 | 17 | M | RS | YES | 55 | **27** | 16 | **1.7** |  | + |  |  |
| 10 | 12 | F | RS | YES | 75 | **22** | 13 | 1.3 |  | + |  |  |
| 11 | 14 | F | RS | YES | 85 | **26** | **27** | 1.1 | + |  |  |  |
| 12 | 12 | M | RS | YES | 58 | **28** | **33** | **2.4** | + | + |  |  |
| 13 | 16 | M | RS | YES | 50 | **24** | **30** | **1.9** | + |  |  |  |
| 14 | 8 | M | RS | YES | N/A | **18** | **21** | 2 |  | + |  |  |
| 15 | 12 | M | RS | YES | 70 | **20** | **19** | **1.9** | + | + |  |  |
| 16 | 16 | M | RS | YES | 50 | **18** | **27** | 1.4 | + |  |  |  |
| 17 | 13 | F | RS | YES | 57 | 14 | **26** | .94 | + |  |  | + |
| 18 | 12 | M | RS | YES | 66 | **23** | 5 | 1 |  | + |  |  |
| 19 | 15 | M | IC | YES | 79 | **25** | **40** | **2.4** | + | + |  | + |

**Abbreviations:** Prolonged Febrile Seizure (PFS), Acute Symptomatic (AS), Remote Symptomatic (RS), Idiopathic/cryptogenic (IC), Unclassified (UNC), Autism Spectrum Disorder (ASD), Attention Deficit Hyperactivity Disorder (ADHD), pervasive developmental disorder not otherwise specified (PDD-NOS), developmental coordination disorder (DCD).
